# Supplementary material for: Activating natural product synthesis using CRISPR interference and activation systems in Streptomyces
Source: Nucleic Acids Res. 2022 Jul 8;50(13):7751–60. doi: 10.1093/nar/gkac556 (PMC9303295; doi:10.1093/nar/gkac556)
Supplement: gkac556_Supplemental_File [file gkac556_supplemental_file.docx]

**Activating natural product synthesis using CRISPR interference and activation systems in Streptomyces.**

Andrea Ameruoso^1^, Maria Claudia Villegas Kcam^1^, Katherine Piper Cohen^1^ and James Chappell^1,2^

1 - Department of BioSciences, Rice University, 6100 Main Street, MS 140, Houston, TX 77005, USA.

2 - Department of Bioengineering, Rice University, 6100 Main Street, MS 142, Houston, TX 77005, USA.

**TABLE OF CONTENTS**

| **Pages 1-4** | Supplementary table 1. List of plasmids used in this study |
| --- | --- |
| **Pages 5-15** | Supplementary table 2. Example DNA plasmid sequences |
| **Page 16** | Supplementary table 3. Promoters used in this study |
| **Pages 16-17** | Supplementary table 4. sgRNA sequences used in this study |
| **Pages 18-19** | Supplementary table 5. Activator domains (ADs) used in this study |
| **Page 20** | Supplementary figure 1. CRISPRi can repress transcription in *Streptomyces*. |
| **Page 21** | Supplementary figure 2. Evaluating the strength of a library of Streptomyces promoters. |
| **Page 22** | Supplementary figure 3. CRISPRi results in decreased fluorescence in the absence of a sgRNA or in the presence of non-targeting sgRNA. |
| **Page 23** | Supplementary figure 4. Evaluating distance-dependent activation patterns of CRISPRa. |
| **Page 24** | Supplementary figure 5. Ion count-based estimation of jadomycin B produced using CRISPRi. |
| **Page 25** | Supplementary figure 6. Additional replicates of the jadomycin B production experiment using CRISPRi. |
| **Page 26** | Supplementary figure 7. Ion count-based estimation of jadomycin B produced using CRISPRa. |
| **Page 27** | Supplementary figure 8. Additional replicates of the jadomycin B production experiment using CRISPRa. |
| **Page 28** | References |

Supplementary table 1. List of all plasmids used in this study. Abbreviations are as follows: R9 = R9 ribosome binding site, ori = origin of replication, specR = spectinomycin resistance gene, apmR = apramycin resistance gene, oriT = origin of transfer, sgRNA = single guide RNA, ds origin = double-stranded origin, bp = base pairs, NT = non-template strand, T = template strand, αNTD = N-terminal domain of the α subunit of RNAP. Promoters: KasO*p, ermE*p, gapdh(EL), rpsL(XC), 57, SP43, SP30, SP20, SP10, SP1. Terminators: T7, λt0, Fd. Origins of replication: pMB1, pUC.

| **Plasmid ID** | **Plasmid features** | **Name** | **Figure(s)** |
| --- | --- | --- | --- |
| pJEC532 | KasO*p - mCherry - T7 - pMB1 ori - SpecR - RP4 oriT - ΦC31 attP site - ΦC31 integrase | mCherry reporter | 1b, 1c, 1d, S1 |
| pJEC533 | gapdh(EL) - mCherry - T7 terminator - pMB1 ori - SpecR - RP4 oriT - ΦC31 attP site - ΦC31 integrase | gapdh(EL)-mCherry | S1 |
| pJEC710 | Fd terminator - gapdh(EL) - lacZ - sgRNA scaffold - λt0 terminator - pUC ori - ApmR - pSG5 replicase - pSG5 ds origin - RP4 oriT | no CRISPR | 1b, 1c, 1d, 2b, 2c, 3d, 3e, S1, S2, S3 |
| pJEC711 | rpsL(XC) - dCas9 - Fd terminator - gapdh(EL) - mCherry sgRNA (+11bp) - sgRNA scaffold - λt0 terminator - pUC ori - ApmR - pSG5 replicase - pSG5 ds origin - RP4 oriT | rpsL(XC)-dCas9/gapdh(EL)-sgRNA | 1b |
| pJEC712 | rpsL(XC) - dCas9 - Fd terminator - SP43 - mCherry sgRNA (+11bp) - sgRNA scaffold - λt0 terminator - pUC ori - ApmR - pSG5 replicase - pSG5 ds origin - RP4 oriT | rpsL(XC)-dCas9/SP43-sgRNA | 1b, 1c |
| pJEC713 | SP1 - RiboJ - R9 - dCas9 - Fd terminator - SP43 - mCherry sgRNA (+11bp) - sgRNA scaffold - λt0 terminator - pUC ori - ApmR - pSG5 replicase - pSG5 ds origin - RP4 oriT | SP1-dCas9/SP43-sgRNA | 1c |
| pJEC714 | SP30 - RiboJ -R9 - dCas9 - Fd terminator - SP43 - mCherry sgRNA (+11bp NT) - sgRNA scaffold - λt0 terminator - pUC ori - ApmR - pSG5 replicase - pSG5 ds origin - RP4 oriT | SP30-dCas9/SP43-sgRNA (+11 NT) | 1c, 1d, S2 |
| pJEC715 | SP43 - RiboJ -R9 - mCherry - T7 terminator - pMB1 ori - SpecR - RP4 oriT - ΦC31 attP site - ΦC31 integrase | SP43-mCherry | S1 |
| pJEC716 | SP30 - RiboJ -R9 - mCherry - T7 terminator - pMB1 ori - SpecR - RP4 oriT - ΦC31 attP site - ΦC31 integrase | SP30-mCherry | S1 |
| pJEC717 | ermE*p - mCherry - T7 terminator - pMB1 ori - SpecR - RP4 oriT - ΦC31 attP site - ΦC31 integrase | ermE*p-mCherry | S1 |
| pJEC718 | SP20 - RiboJ -R9 - mCherry - T7 terminator - pMB1 ori - SpecR - RP4 oriT - ΦC31 attP site - ΦC31 integrase | SP20-mCherry | S1 |
| pJEC719 | SP10 - RiboJ -R9 - mCherry - T7 terminator - pMB1 ori - SpecR - RP4 oriT - ΦC31 attP site - ΦC31 integrase | SP10-mCherry | 2b, 2c, S1 |
| pJEC720 | rpsL(XC) - mCherry - T7 terminator - pMB1 ori - SpecR - RP4 oriT - ΦC31 attP site - ΦC31 integrase | rpsL(XC)-mCherry | S1 |
| pJEC721 | 57 - mCherry - T7 terminator - pMB1 ori - SpecR - RP4 oriT - ΦC31 attP site - ΦC31 integrase | 57-mCherry | S1 |
| pJEC722 | SP1 - RiboJ -R9 - mCherry - T7 terminator - pMB1 ori - SpecR - RP4 oriT - ΦC31 attP site - ΦC31 integrase | SP1-mCherry | S1 |
| pJEC723 | SP30 - RiboJ -R9 - dCas9 - Fd terminator - SP43 - non-coding genomic region sgRNA #1 - sgRNA scaffold - λt0 terminator - pUC ori - ApmR - pSG5 replicase - pSG5 ds origin - RP4 oriT | CRISPRi with sgRNA binding to a genomic region | 1d,S2 |
| pJEC724 | SP30 - RiboJ -R9 - dCas9 - Fd terminator - SP43 - non-coding genomic region sgRNA #2 - sgRNA scaffold - λt0 terminator - pUC ori - ApmR - pSG5 replicase - pSG5 ds origin - RP4 oriT | CRISPRi with sgRNA binding to a genomic region | S2 |
| pJEC725 | SP30 - RiboJ -R9 - dCas9 - Fd terminator - SP43 - no-match sgRNA #2 - sgRNA scaffold - λt0 terminator - pUC ori - ApmR - pSG5 replicase - pSG5 ds origin - RP4 oriT | SP30-dCas9/SP43-sgRNA off-target w/o binding site | S2 |
| pJEC726 | SP30 - RiboJ -R9 - dCas9 - Fd terminator - SP43 - lacZ - sgRNA scaffold - λt0 terminator - pUC ori - ApmR - pSG5 replicase - pSG5 ds origin - RP4 oriT | SP30-dCas9/SP43-sgRNA scaffold (i.e. no sgRNA control) | S2 |
| pJEC727 | SP30 - RiboJ -R9 - dCas9 - Fd terminator - SP43 - mCherry sgRNA (+123bp NT) - sgRNA scaffold - λt0 terminator - pUC ori - ApmR - pSG5 replicase - pSG5 ds origin - RP4 oriT | SP30-dCas9/SP43-sgRNA (+123 NT) | 1d |
| pJEC728 | SP30 - RiboJ -R9 - dCas9 - Fd terminator - SP43 - mCherry sgRNA (+230bp NT) - sgRNA scaffold - λt0 terminator - pUC ori - ApmR - pSG5 replicase - pSG5 ds origin - RP4 oriT | SP30-dCas9/SP43-sgRNA (+230 NT) | 1d |
| pJEC729 | SP30 - RiboJ -R9 - dCas9 - Fd terminator - SP43 - mCherry sgRNA (+531bp NT) - sgRNA scaffold - λt0 terminator - pUC ori - ApmR - pSG5 replicase - pSG5 ds origin - RP4 oriT | SP30-dCas9/SP43-sgRNA (+531 NT) | 1d |
| pJEC730 | SP30 - RiboJ -R9 - dCas9 - Fd terminator - SP43 - mCherry sgRNA (+623bp NT) - sgRNA scaffold - λt0 terminator - pUC ori - ApmR - pSG5 replicase - pSG5 ds origin - RP4 oriT | SP30-dCas9/SP43-sgRNA (+623 NT) | 1d |
| pJEC731 | SP30 - RiboJ -R9 - dCas9 - Fd terminator - SP43 - mCherry sgRNA (+9bp T) - sgRNA scaffold - λt0 terminator - pUC ori - ApmR - pSG5 replicase - pSG5 ds origin - RP4 oriT | SP30-dCas9/SP43-sgRNA (+9 T) | 1d |
| pJEC732 | SP30 - RiboJ -R9 - dCas9 - Fd terminator - SP43 - mCherry sgRNA (+112bp T) - sgRNA scaffold - λt0 terminator - pUC ori - ApmR - pSG5 replicase - pSG5 ds origin - RP4 oriT | SP30-dCas9/SP43-sgRNA (+112 T) | 1d |
| pJEC733 | SP30 - RiboJ -R9 - dCas9 - Fd terminator - SP43 - mCherry sgRNA (+223bp T) - sgRNA scaffold - λt0 terminator - pUC ori - ApmR - pSG5 replicase - pSG5 ds origin - RP4 oriT | SP30-dCas9/SP43-sgRNA (+223 T) | 1d |
| pJEC734 | SP30 - RiboJ -R9 - dCas9 - Fd terminator - SP43 - mCherry sgRNA (+540bp T) - sgRNA scaffold - λt0 terminator - pUC ori - ApmR - pSG5 replicase - pSG5 ds origin - RP4 oriT | SP30-dCas9/SP43-sgRNA (+540 T) | 1d |
| pJEC735 | SP30 - RiboJ -R9 - dCas9 - Fd terminator - SP43 - mCherry sgRNA (+627bp T) - sgRNA scaffold - λt0 terminator - pUC ori - ApmR - pSG5 replicase - pSG5 ds origin - RP4 oriT | SP30-dCas9/SP43-sgRNA (+627 T) | 1d |
| pJEC736 | PAM region - SP10 - RiboJ -R9 - mCherry - T7 terminator - pMB1 ori - SpecR - RP4 oriT - ΦC31 attP site - ΦC31 integrase | CRISPRa reporter #1 | 2b, 2c, S3 |
| pJEC737 | SP30 - RiboJ -R9 - dCas9 - XTEN - αNTD - Fd terminator - SP43 - non-coding genomic region sgRNA #1 - sgRNA scaffold - λt0 terminator - pUC ori - ApmR - pSG5 replicase - pSG5 ds origin - RP4 oriT | SP30-dCas9-αNTD/off-target | 2b |
| pJEC738 | SP30 - RiboJ -R9 - dCas9 - XTEN - αNTD - Fd terminator - SP43 - CRISPRa sgRNA (82bp T) - sgRNA scaffold - λt0 terminator - pUC ori - ApmR - pSG5 replicase - pSG5 ds origin - RP4 oriT | SP30-dCas9-αNTD/sgRNA -82 T | 2b |
| pJEC739 | SP30 - RiboJ -R9 - dCas9 - XTEN - αNTD - Fd terminator - SP43 - CRISPRa sgRNA (83bp NT) - sgRNA scaffold - λt0 terminator - pUC ori - ApmR - pSG5 replicase - pSG5 ds origin - RP4 oriT | SP30-dCas9-αNTD/sgRNA -83 NT | 2b, 2c, S3 |
| pJEC740 | SP30 - RiboJ -R9 - dCas9 - XTEN - ω - Fd terminator - SP43 - non-coding genomic region sgRNA #1 - sgRNA scaffold - λt0 terminator - pUC ori - ApmR - pSG5 replicase - pSG5 ds origin - RP4 oriT | SP30-dCas9-ω/off-target | 2b |
| pJEC741 | SP30 - RiboJ -R9 - dCas9 - XTEN - ω - Fd terminator - SP43 - CRISPRa sgRNA (83bp NT) - sgRNA scaffold - λt0 terminator - pUC ori - ApmR - pSG5 replicase - pSG5 ds origin - RP4 oriT | SP30-dCas9-ω/sgRNA -83 NT | 2b |
| pJEC742 | SP30 - RiboJ -R9 - dCas9 - XTEN - ω - Fd terminator - SP43 - CRISPRa sgRNA (82bp T) - sgRNA scaffold - λt0 terminator - pUC ori - ApmR - pSG5 replicase - pSG5 ds origin - RP4 oriT | SP30-dCas9-ω/sgRNA -82 T | 2b |
| pJEC743 | SP30 - RiboJ -R9 - dCas9 - XTEN - RbpA - Fd terminator - SP43 - non-coding genomic region sgRNA #1 - sgRNA scaffold - λt0 terminator - pUC ori - ApmR - pSG5 replicase - pSG5 ds origin - RP4 oriT | SP30-dCas9-RbpA/off-target | 2b |
| pJEC744 | SP30 - RiboJ -R9 - dCas9 - XTEN - RbpA - Fd terminator - SP43 - CRISPRa sgRNA (83bp NT) - sgRNA scaffold - λt0 terminator - pUC ori - ApmR - pSG5 replicase - pSG5 ds origin - RP4 oriT | SP30-dCas9-RbpA/sgRNA -83 NT | 2b |
| pJEC745 | SP30 - RiboJ -R9 - dCas9 - XTEN - RbpA - Fd terminator - SP43 - CRISPRa sgRNA (82bp T) - sgRNA scaffold - λt0 terminator - pUC ori - ApmR - pSG5 replicase - pSG5 ds origin - RP4 oriT | SP30-dCas9-RbpA/sgRNA -82 T | 2b |
| pJEC746 | PAM region + 5bp - SP10 - RiboJ -R9 - mCherry - T7 terminator - pMB1 ori - SpecR - RP4 oriT - ΦC31 attP site - ΦC31 integrase | CRISPRa reporter #2 (PAMs shifted by 5bp) | 2c, S3 |
| pJEC747 | SP30 - RiboJ -R9 - dCas9 - XTEN - αNTD - Fd terminator - SP43 - CRISPRa sgRNA (73bp NT) - sgRNA scaffold - λt0 terminator - pUC ori - ApmR - pSG5 replicase - pSG5 ds origin - RP4 oriT | SP30-dCas9-αNTD/sgRNA -73 NT | 2c, S3 |
| pJEC748 | SP30 - RiboJ -R9 - dCas9 - XTEN - αNTD - Fd terminator - SP43 - CRISPRa sgRNA (93bp NT) - sgRNA scaffold - λt0 terminator - pUC ori - ApmR - pSG5 replicase - pSG5 ds origin - RP4 oriT | SP30-dCas9-αNTD/sgRNA -93 NT | 2c, S3 |
| pJEC749 | SP30 - RiboJ -R9 - dCas9 - Fd terminator - SP43 - CRISPRi jadR2 sgRNA (+44bp NT) - sgRNA scaffold - λt0 terminator - pUC ori - ApmR - pSG5 replicase - pSG5 ds origin - RP4 oriT | jadR2 CRISPRi | 3d |
| pJEC750 | SP30 - RiboJ -R9 - dCas9 - XTEN - αNTD - Fd terminator - SP43 - CRISPRa jadJ sgRNA (-73bp NT) - sgRNA scaffold - λt0 terminator - pUC ori - ApmR - pSG5 replicase - pSG5 ds origin - RP4 oriT | jadJ CRISPRa | 3e |

Supplementary table 2. Example DNA plasmid sequences.

| **Name and features** | **DNA sequence** |
| --- | --- |
| CRISPRi reporter  (Promoter-RBS-mCherry-Terminator-E.coli ori-SpecR-oriT-attP site-phiC31 integrase) | CGAGACACCCGGGAAGCCTGATCTACGTCTGTCGAGAAGTTTCTGATCGATGACACTCGTTCGTTCACACGTTGCAGCAGAGTACTTGTTCACATTCGAACGGTCTCTGCTTTGACAACATGCTGTGCGGTGTTGTAAAGTCGTGGCCAGGAGAATACGACAGCGTGCAGGACTGGGGGAGTGCGCATATGGTCTCCAAGGGCGAGGAGGACAACATGGCCATCATCAAGGAGTTCATGCGCTTCAAGGTCCACATGGAGGGCTCCGTCAACGGGCACGAGTTCGAGATCGAGGGCGAGGGGGAGGGCCGGCCGTACGAGGGCACCCAGACCGCCAAGCTGAAGGTGACCAAGGGCGGCCCCCTCCCGTTCGCCTGGGACATCCTCTCCCCCCAGTTCATGTACGGCTCGAAGGCCTACGTCAAGCACCCGGCCGACATCCCGGACTACCTGAAGCTCTCGTTCCCGGAGGGGTTCAAGTGGGAGCGGGTCATGAACTTCGAGGACGGCGGCGTCGTCACCGTCACCCAGGACAGCTCCCTGCAGGACGGCGAGTTCATCTACAAGGTCAAGCTGCGGGGCACGAACTTCCCGAGCGACGGCCCCGTGATGCAGAAGAAGACGATGGGCTGGGAAGCGTCCTCGGAGCGCATGTACCCGGAGGACGGCGCCCTCAAGGGCGAGATCAAGCAGCGCCTGAAGCTGAAGGACGGCGGCCACTACGACGCCGAAGTCAAGACGACGTACAAGGCCAAGAAGCCGGTGCAGCTCCCGGACGCCTACAACGTGAACATCAAGCTCGACATCACCTCGCACAACGAGGACTACACGATCGTGGAGCAGTACGAGCGCGCCGAGGGCCGGCACTCGACCGGCGGCATGGACGAGCTGTACAAGTGAGCCGTTCGCGCCGCCCCGGCTCGCATCGTCCCCGACCCGTCAACCTCATCCGCAAGGAGTCTCTAGAGGATCCGCGGCCGCGCGCGATATCGAATTCCTCGAGTAACTAGCATAACCCCTTGGGGCCTCTAAACGGGTCTTGAGGGGTTTTTTGTTAAGCCGAACAGGAAGCACAGCTCCTACTGAACATGTGAGCAAAAGGCCAGCAAAAGGCCAGGAACCGTAAAAAGGCCGCGTTGCTGGCGTTTTTCCATAGGCTCCGCCCCCCTGACGAGCATCACAAAAATCGACGCTCAAGTCAGAGGTGGCGAAACCCGACAGGACTATAAAGATACCAGGCGTTTCCCCCTGGAAGCTCCCTCGTGCGCTCTCCTGTTCCGACCCTGCCGCTTACCGGATACCTGTCCGCCTTTCTCCCTTCGGGAAGCGTGGCGCTTTCTCATAGCTCACGCTGTAGGTATCTCAGTTCGGTGTAGGTCGTTCGCTCCAAGCTGGGCTGTGTGCACGAACCCCCCGTTCAGCCCGACCGCTGCGCCTTATCCGGTAACTATCGTCTTGAGTCCAACCCGGTAAGACACGACTTATCGCCACTGGCAGCAGCCACTGGTAACAGGATTAGCAGAGCGAGGTATGTAGGCGGTGCTACAGAGTTCTTGAAGTGGTGGCCTAACTACGGCTACACTAGAAGAACAGTATTTGGTATCTGCGCTCTGCTGAAGCCAGTTACCTTCGGAAAAAGAGTTGGTAGCTCTTGATCCGGCAAACAAACCACCGCTGGTAGCGGTGGTTTTTTTGTTTGCAAGCAGCAGATTACGCGCAGAAAAAAAGGATCTCAAGAAGATCCTTTGATCTTTTCTACGGGGTCTGACGCTCAGTGGAACGAAAACTCACGTTAAGGGATTTTGGTCATGAGATTATCAAAAAGGATCTTCACCTAGATCCTTTTGGTTCATGTGCAGCTCCATCAGCAAAAGGGGATGATAAGTTTATCACCACCGACTATTTGCAACAGTGCCGTTGATCGTGCTATGATCGACTGATGTCATCAGCGGTGGAGTGCAATGTCATGCGCTCACGCAACTGGTCCAGAACCTTGACCGAACGCAGCGGTGGTAACGGCGCAGTGGCGGTTTTCATGGCTTGTTATGACTGTTTTTTTGGGGTACAGTCTATGCCTCGGGCATCCAAGCAGCAAGCGCGTTACGCCGTGGGTCGATGTTTGATGTTATGGAGCAGCAACGATGTTACGCAGCAGGGCAGTCGCCCTAAAACAAAGTTAAACATC ATGAGGGAAGCGGTGATCGCCGAAGTATCGACTCAACTATCAGAGGTAGTTGGCGTCATCGAGCGCCATCTCGAACCGACGTTGCTGGCCGTACATTTGTACGGCTCCGCAGTGGATGGCGGCCTGAAGCCACACAGTGATATTGATTTGCTGGTTACGGTGACCGTAAGGCTTGATGAAACAACGCGGCGAGCTTTGATCAACGACCTTTTGGAAACTTCGGCTTCCCCTGGAGAGAGCGAGATTCTCCGCGCTGTAGAAGTCACCATTGTTGTGCACGACGACATCATTCCGTGGCGTTATCCAGCTAAGCGCGAACTGCAATTTGGAGAATGGCAGCGCAATGACATTCTTGCAGGTATCTTCGAGCCAGCCACGATCGACATTGATCTGGCTATCTTGCTGACAAAAGCAAGAGAACATAGCGTTGCCTTGGTAGGTCCAGCGGCGGAGGAACTCTTTGATCCGGTTCCTGAACAGGATCTATTTGAGGCGCTAAATGAAACCTTAACGCTATGGAACTCGCCGCCCGACTGGGCTGGCGATGAGCGAAATGTAGTGCTTACGTTGTCCCGCATTTGGTACAGCGCAGTAACCGGCAAAATCGCGCCGAAGGATGTCGCTGCCGACTGGGCAATGGAGCGCCTGCCGGCCCAGTATCAGCCCGTCATACTTGAAGCTAGACAGGCTTATCTTGGACAAGAAGAAGATCGCTTGGCCTCGCGCGCAGATCAGTTGGAAGAATTTGTCCACTACGTGAAAGGCGAGATCACCAAGGTAGTCGGCAAATAATTGTCTTTCTTCAGCTCGCTGATGATATGCCTTCCTGGTTGGCTTGGTTTCATCAGCCATCCGCTTGCCCTCATCTGTTACGCCGGCGGTAGCCGGCCAGCCTCGCAGAGCAGGATTCCCGTTGAGCACCGCCAGGTGCGAATAAGGGACAGTGAAGAAGGAACACCCGCTCGCGGGTGGGCCTACTTCACCTATCCTGCCCGGCTGACGCCGTTGGATACACCAAGGAAAGTCTACACGAACCCTTTGGCAAAATCCTGTATATCGTGCGAAAAAGGATGGATATACCGAAAAAATCGCTATAATGACCCCGAAGCAGGGTTATGCAGCGGAAAAGATCCGTCGACCTGCAGGCATGCAAGCTCTAGCGATTCCAGACGTCCCGAAGGCGTGGCGCGGCTTCCCCGTGCCGGAGCAATCGCCCTGGGTGGGTTACACGACGCCCCTCTATGGCCCGTACTGACGGACACACCGAAGCCCCGGCGGCAACCCTCAGCGGATGCCCCGGGGCTTCACGTTTTCCCAGGTCAGAAGCGGTTTTCGGGAGTAGTGCCCCAACTGGGGTAACCTTTGAGTTCTCTCAGTTGGGGGCGTAGGGTCGCCGACATGACACAAGGGGTTGTGACCGGGGTGGACACGTACGCGGGTGCTTACGACCGTCAGTCGCGCGAGCGCGAGAATTCGAGCGCAGCAAGCCCAGCGACACAGCGTAGCGCCAACGAAGACAAGGCGGCCGACCTTCAGCGCGAAGTCGAGCGCGACGGGGGCCGGTTCAGGTTCGTCGGGCATTTCAGCGAAGCGCCGGGCACGTCGGCGTTCGGGACGGCGGAGCGCCCGGAGTTCGAACGCATCCTGAACGAATGCCGCGCCGGGCGGCTCAACATGATCATTGTCTATGACGTGTCGCGCTTCTCGCGCCTGAAGGTCATGGACGCGATTCCGATTGTCTCGGAATTGCTCGCCCTGGGCGTGACGATTGTTTCCACTCAGGAAGGCGTCTTCCGGCAGGGAAACGTCATGGACCTGATTCACCTGATTATGCGGCTCGACGCGTCGCACAAAGAATCTTCGCTGAAGTCGGCGAAGATTCTCGACACGAAGAACCTTCAGCGCGAATTGGGCGGGTACGTCGGCGGGAAGGCGCCTTACGGCTTCGAGCTTGTTTCGGAGACGAAGGAGATCACGCGCAACGGCCGAATGGTCAATGTCGTCATCAACAAGCTTGCGCACTCGACCACTCCCCTTACCGGACCCTTCGAGTTCGAGCCCGACGTAATCCGGTGGTGGTGGCGTGAGATCAAGACGCACAAACACCTTCCCTTCAAGCCGGGCAGTCAAGCCGCCATTCACCCGGGCAGCATCACGGGGCTTTGTAAGCGCATGGACGCTGACGCCGTGCCGACCCGGGGCGAGACGATTGGGAAGAAGACCGCTTCAAGCGCCTGGGACCCGGCAACCGTTATGCGAATCCTTCGGGACCCGCGTATTGCGGGCTTCGCCGCTGAGGTGATCTACAAGAAGAAGCCGGACGGCACGCCGACCACGAAGATTGAGGGTTACCGCATTCAGCGCGACCCGATCACGCTCCGGCCGGTCGAGCTTGATTGCGGACCGATCATCGAGCCCGCTGAGTGGTATGAGCTTCAGGCGTGGTTGGACGGCAGGGGGCGCGGCAAGGGGCTTTCCCGGGGGCAAGCCATTCTGTCCGCCATGGACAAGCTGTACTGCGAGTGTGGCGCCGTCATGACTTCGAAGCGCGGGGAAGAATCGATCAAGGACTCTTACCGCTGCCGTCGCCGGAAGGTGGTCGACCCGTCCGCACCTGGGCAGCACGAAGGCACGTGCAACGTCAGCATGGCGGCACTCGACAAGTTCGTTGCGGAACGCATCTTCAACAAGATCAGGCACGCCGAAGGCGACGAAGAGACGTTGGCGCTTCTGTGGGAAGCCGCCCGACGCTTCGGCAAGCTCACTGAGGCGCCTGAGAAGAGCGGCGAACGGGCGAACCTTGTTGCGGAGCGCGCCGACGCCCTGAACGCCCTTGAAGAGCTGTACGAAGACCGCGCGGCAGGCGCGTACGACGGACCCGTTGGCAGGAAGCACTTCCGGAAGCAACAGGCAGCGCTGACGCTCCGGCAGCAAGGGGCGGAAGAGCGGCTTGCCGAACTTGAAGCCGCCGAAGCCCCGAAGCTTCCCCTTGACCAATGGTTCCCCGAAGACGCCGACGCTGACCCGACCGGCCCTAAGTCGTGGTGGGGGCGCGCGTCAGTAGACGACAAGCGCGTGTTCGTCGGGCTCTTCGTAGACAAGATCGTTGTCACGAAGTCGACTACGGGCAGGGGGCAGGGAACGCCCATCGAGAAGCGCGCTTCGATCACGTGGGCGAAGCCGCCGACCGACGACGACGAAGACGACGCCCAGGACGGCACGGAAGACGTAGCGGCGTAG |
| CRISPRi  (Promoter-RiboJ-RBS-dCas9-terminator-promoter-sgRNA targeting sequence-sgRNA scaffold-terminator-ori-AprR-pSG5 rep-pSG5 ds-oriT) | TGTTCACATTCGAACCGTCTCTGCTTTGACATCGTGTGGCGCTTGGGTGTAAAGTCGTGGCCATTAAACAAAATTATTTGTAGAGGCTGTTTCGTCCTCACGGACTCATCAGACCGGAAAGCACATCCGGTGACAGCTAACTACGAAGGGGAGTCAGTATGGACAAGAAGTACAGCATCGGCCTGGCCATCGGCACCAACAGCGTGGGCTGGGCGGTCATCACCGACGAGTACAAGGTCCCCTCCAAGAAGTTCAAGGTCCTGGGCAACACCGACCGGCACTCGATCAAGAAGAACCTGATCGGCGCCCTGCTCTTCGACAGCGGCGAAACCGCCGAGGCGACCCGCCTGAAGCGGACCGCCCGTCGCCGCTACACCCGGCGCAAGAACCGCATCTGCTACCTGCAGGAGATCTTCTCCAACGAGATGGCCAAGGTCGACGACTCGTTCTTCCACCGGCTCGAGGAGAGCTTCCTGGTGGAGGAGGACAAGAAGCACGAGCGCCACCCGATCTTCGGCAACATCGTCGACGAGGTGGCCTACCACGAGAAGTACCCCACCATCTACCACCTCCGCAAGAAGCTGGTCGACTCGACCGACAAGGCGGACCTGCGGCTCATCTACCTGGCCCTCGCGCACATGATCAAGTTCCGCGGCCACTTCCTCATCGAGGGCGACCTGAACCCGGACAACTCCGACGTCGACAAGCTCTTCATCCAGCTGGTGCAGACCTACAACCAGCTGTTCGAGGAGAACCCCATCAACGCCAGCGGCGTCGACGCCAAGGCGATCCTCTCCGCGCGCCTGAGCAAGTCCCGGCGCCTGGAGAACCTCATCGCCCAGCTGCCGGGCGAGAAGAAGAACGGCCTCTTCGGCAACCTGATCGCGCTGTCGCTCGGCCTGACCCCCAACTTCAAGAGCAACTTCGACCTGGCCGAGGACGCGAAGCTCCAGCTGTCCAAGGACACCTACGACGACGACCTGGACAACCTGCTCGCCCAGATCGGCGACCAGTACGCGGACCTCTTCCTGGCCGCGAAGAACCTCTCGGACGCCATCCTGCTCAGCGACATCCTGCGGGTCAACACCGAGATCACCAAGGCCCCGCTGTCGGCGAGCATGATCAAGCGGTACGACGAGCACCACCAGGACCTGACCCTGCTCAAGGCCCTCGTGCGCCAGCAGCTGCCCGAGAAGTACAAGGAGATCTTCTTCGACCAGTCCAAGAACGGCTACGCCGGCTACATCGACGGCGGCGCGTCGCAGGAGGAGTTCTACAAGTTCATCAAGCCGATCCTGGAGAAGATGGACGGCACCGAGGAGCTGCTCGTCAAGCTGAACCGCGAGGACCTGCTCCGCAAGCAGCGGACCTTCGACAACGGCTCCATCCCGCACCAGATCCACCTGGGCGAGCTCCACGCCATCCTCCGGCGCCAGGAGGACTTCTACCCCTTCCTGAAGGACAACCGCGAGAAGATCGAGAAGATCCTGACCTTCCGGATCCCGTACTACGTCGGCCCCCTGGCCCGCGGCAACTCCCGGTTCGCGTGGATGACCCGGAAGTCGGAGGAAACCATCACCCCGTGGAACTTCGAGGAGGTCGTGGACAAGGGCGCCTCCGCGCAGTCGTTCATCGAGCGCATGACCAACTTCGACAAGAACCTCCCGAACGAGAAGGTCCTGCCCAAGCACAGCCTGCTCTACGAGTACTTCACCGTGTACAACGAGCTGACCAAGGTCAAGTACGTGACCGAGGGCATGCGGAAGCCGGCCTTCCTGTCCGGCGAGCAGAAGAAGGCGATCGTCGACCTGCTCTTCAAGACCAACCGCAAGGTCACCGTGAAGCAGCTGAAGGAGGACTACTTCAAGAAGATCGAGTGCTTCGACTCCGTCGAGATCTCGGGCGTGGAGGACCGCTTCAACGCCTCCCTGGGCACCTACCACGACCTGCTCAAGATCATCAAGGACAAGGACTTCCTCGACAACGAGGAGAACGAGGACATCCTGGAGGACATCGTCCTCACCCTGACCCTCTTCGAGGACCGCGAGATGATCGAGGAGCGGCTCAAGACCTACGCCCACCTGTTCGACGACAAGGTGATGAAGCAGCTGAAGCGGCGCCGGTACACCGGCTGGGGCCGCCTCTCCCGGAAGCTGATCAACGGCATCCGGGACAAGCAGAGCGGCAAGACCATCCTGGACTTCCTCAAGTCCGACGGCTTCGCCAACCGCAACTTCATGCAGCTCATCCACGACGACTCGCTGACCTTCAAGGAGGACATCCAGAAGGCCCAGGTGTCCGGCCAGGGCGACAGCCTCCACGAGCACATCGCCAACCTGGCGGGCTCCCCGGCGATCAAGAAGGGCATCCTCCAGACCGTCAAGGTCGTGGACGAGCTGGTCAAGGTGATGGGCCGCCACAAGCCCGAGAACATCGTGATCGAGATGGCCCGGGAGAACCAGACCACCCAGAAGGGCCAGAAGAACTCCCGCGAGCGGATGAAGCGCATCGAGGAGGGCATCAAGGAGCTCGGCTCGCAGATCCTGAAGGAGCACCCGGTCGAGAACACCCAGCTCCAGAACGAGAAGCTGTACCTCTACTACCTGCAGAACGGCCGCGACATGTACGTGGACCAGGAGCTCGACATCAACCGGCTGAGCGACTACGACGTCGACGCCATCGTGCCGCAGTCCTTCCTGAAGGACGACTCGATCGACAACAAGGTCCTGACCCGCTCCGACAAGAACCGGGGCAAGTCCGACAACGTGCCCTCGGAGGAGGTCGTGAAGAAGATGAAGAACTACTGGCGCCAGCTGCTCAACGCCAAGCTCATCACCCAGCGCAAGTTCGACAACCTGACCAAGGCCGAGCGGGGCGGCCTGTCGGAGCTCGACAAGGCGGGCTTCATCAAGCGCCAGCTCGTCGAAACCCGGCAGATCACCAAGCACGTGGCCCAGATCCTGGACAGCCGGATGAACACCAAGTACGACGAGAACGACAAGCTGATCCGCGAGGTCAAGGTGATCACCCTCAAGAGCAAGCTGGTGTCCGACTTCCGCAAGGACTTCCAGTTCTACAAGGTCCGGGAGATCAACAACTACCACCACGCCCACGACGCGTACCTGAACGCCGTCGTGGGCACCGCGCTGATCAAGAAGTACCCGAAGCTGGAGTCCGAGTTCGTCTACGGCGACTACAAGGTCTACGACGTGCGCAAGATGATCGCCAAGTCGGAGCAGGAGATCGGCAAGGCCACCGCGAAGTACTTCTTCTACAGCAACATCATGAACTTCTTCAAGACCGAGATCACCCTGGCCAACGGCGAGATCCGCAAGCGGCCCCTGATCGAAACCAACGGCGAAACCGGCGAGATCGTCTGGGACAAGGGCCGCGACTTCGCCACCGTCCGGAAGGTGCTGTCCATGCCGCAGGTCAACATCGTCAAGAAAACCGAGGTGCAGACCGGCGGCTTCAGCAAGGAGTCCATCCTCCCCAAGCGCAACTCGGACAAGCTGATCGCCCGGAAGAAGGACTGGGACCCGAAGAAGTACGGCGGCTTCGACAGCCCCACCGTCGCCTACTCCGTGCTGGTCGTGGCGAAGGTCGAGAAGGGCAAGAGCAAGAAGCTGAAGTCCGTGAAGGAGCTGCTCGGCATCACCATCATGGAGCGCTCCTCGTTCGAGAAGAACCCGATCGACTTCCTGGAGGCCAAGGGCTACAAGGAGGTCAAGAAGGACCTCATCATCAAGCTGCCCAAGTACTCGCTGTTCGAGCTCGAGAACGGCCGCAAGCGGATGCTCGCCAGCGCGGGCGAGCTGCAGAAGGGCAACGAGCTGGCCCTCCCGTCCAAGTACGTCAACTTCCTGTACCTCGCGTCCCACTACGAGAAGCTGAAGGGCTCGCCCGAGGACAACGAGCAGAAGCAGCTCTTCGTGGAGCAGCACAAGCACTACCTGGACGAGATCATCGAGCAGATCTCGGAGTTCAGCAAGCGGGTCATCCTGGCCGACGCGAACCTCGACAAGGTGCTGTCCGCCTACAACAAGCACCGCGACAAGCCGATCCGGGAGCAGGCGGAGAACATCATCCACCTGTTCACCCTCACCAACCTGGGCGCCCCCGCCGCGTTCAAGTACTTCGACACCACCATCGACCGCAAGCGGTACACCAGCACCAAGGAGGTCCTCGACGCGACCCTGATCCACCAGTCCATCACCGGCCTGTACGAAACCCGCATCGACCTCTCCCAGCTCGGCGGCGACTGAGAATTCAGATCTACGCGTTCCCCGCAAAAGCGGCCTTTGACTCCCTGCAAGCCTCAGCGACCGAATATATCGGTTATGCGTGGGCGATGGTTGTTGTCATTGTCGGCGCAACTATCGGTATCAAGCTGTTTAAGAAATTCACCTCGAAAGCAAGCTGATAAACCGATACAATTAAAGGCTCCTTTTGGAGCCTTTTTTTGACAGCGTGCAGGACTGGGGGAGTTATGTTCACATTCGAACCGTCTCTGCTTTGACACGGACAAGCGCTATGGTGTAAAGTCGTGGCCACATGTTGTCCTCCTCGCCCTGTTTTAGAGCTAGAAATAGCAAGTTAAAATAAGGCTAGTCCGTTATCAACTTGAAAAAGTGGCACCGAGTCGGTGCTTTTTACTCCATCTGGATTTGTTCAGAACGCTCGGTTGCCGCCGGGCGTTTTTTATCTAGAGGCCAGGAACCGTAAAAAGGCCGCGTTGCTGGCGTTTTTCCATAGGCTCCGCCCCCCTGACGAGCATCACAAAAATCGACGCTCAAGTCAGAGGTGGCGAAACCCGACAGGACTATAAAGATACCAGGCGTTTCCCCCTGGAAGCTCCCTCGTGCGCTCTCCTGTTCCGACCCTGCCGCTTACCGGATACCTGTCCGCCTTTCTCCCTTCGGGAAGCGTGGCGCTTTCTCATAGCTCACGCTGTAGGTATCTCAGTTCGGTGTAGGTCGTTCGCTCCAAGCTGGGCTGTGTGCACGACCCCCCCGTTCAGCCCGACCGCTGCGCCTTATCCGGTAACTATCGTCTTGAGTCCAACCCGGTAAGACACGACTTATCGCCACTGGCAGCAGCCACTGGTAACAGGATTAGCAGAGCGAGGTATGTAGGCGGTGCTACAGAGTTCTTGAAGTGGTGGCCTAACTACGGCTACACTAGAAGAACAGTATTTGGTATCTGCGCTCTGCTGAAGCCAGTTACCTTCGGAAAAAGAGTTGGTAGCTCTTGATCCGGCAAACAAACCACCGCTGGTAGCGGTGGTTTTTTTGTTTGCAAGCAGCAGATTACGCGCAGAAAAAAAGGATCTCAAGAAGATCCTTTGATCTTTTCTACGGGGTCTGACGCTCAGTGGAACGAAAACTCACGTTAAGGGATTTTGGTCATGAGATTATCAAAAAGGATCTTCACCTAGATCCTTTTGGTTCATGTGCAGCTCCATCAGCAAAAGGGGATGATAAGTTTATCACCACCGACTATTTGCAACAGTGCCGTTGATCGTGCTATGATCGACTGATGTCATCAGCGGTGGAGTGCAATGTCGTGCAATACGAATGGCGAAAAGCCGAGCTCATCGGTCAGCTTCTCAACCTTGGGGTTACCCCCGGCGGTGTGCTGCTGGTCCACAGCTCCTTCCGTAGCGTCCGGCCCCTCGAAGATGGGCCACTTGGACTGATCGAGGCCCTGCGTGCTGCGCTGGGTCCGGGAGGGACGCTCGTCATGCCCTCGTGGTCAGGTCTGGACGACGAGCCGTTCGATCCTGCCACGTCGCCCGTTACACCGGACCTTGGAGTTGTCTCTGACACATTCTGGCGCCTGCCAAATGTAAAGCGCAGCGCCCATCCATTTGCCTTTGCGGCAGCGGGGCCACAGGCAGAGCAGATCATCTCTGATCCATTGCCCCTGCCACCTCACTCGCCTGCAAGCCCGGTCGCCCGTGTCCATGAACTCGATGGGCAGGTACTTCTCCTCGGCGTGGGACACGATGCCAACACGACGCTGCATCTTGCCGAGTTGATGGCAAAGGTTCCCTATGGGGTGCCGAGACACTGCACCATTCTTCAGGATGGCAAGTTGGTACGCGTCGATTATCTCGAGAATGACCACTGCTGTGAGCGCTTTGCCTTGGCGGACAGGTGGCTCAAGGAGAAGAGCCTTCAGAAGGAAGGTCCAGTCGGTCATGCCTTTGCTCGGTTGATCCGCTCCCGCGACATTGTGGCGACAGCCCTGGGTCAACTGGGCCGAGATCCGTTGATCTTCCTGCATCCGCCAGAGGCGGGATGCGAAGAATGCGATGCCGCTCGCCAGTCGATTGGCTGAGCTCATGAGCGGAGAACGAGATGACGTTGGAGGGGCAAGGTCGCGCTGATTGCTGGGGCAACACGTGGAGCGGATCGGGGATTGTCTTTCTTCAGCTCGCTGATGATATGCTGACGCTCAATGCCGTTTGGCCTCCGACTAACGAAAATCCCGCATTTGGACGGCTGATCCGATTGGCACGGCGGACGGCGAATGGCGGAGCAGACGCTCGTCCGGGGGCAATGAGATATGAAAAAGCCTGAACTCACCGCGACGTATCGATGTCGAGGTTCCTCAGGGGAGCCACCCCAGAGAAGCCCTCGGAGCTGAGCGGAGCTATTTCCAAAGCCATGCCAGCTAGAGACAGTGCACACCGCCAAGCATCTGCAAAACCCTCGCCTGGAGGGAAAGTGCAATGTACGTACGCTGGTTTCCCTCCAGAAAGGGATTCTGCGGCTTCTCAACTTTGGAAGAAGAGGCGGAACGAACTGCTGTTTCAGCCTACTCATGTGAGGAGGCTGGTCCTTTACCCTGATGCCCGAGGGCATCAGGGTAAAGGACCAGCCTCGCCAACTAGGAGCGCTACCAAGGCGAACACAACCCAGGACTTGGTAGAACCCTTCGCCGGAGAAGTTCAGACTCATCGGCATGAGCACCCCTCGCCGCGCACTCGGCACCGGTCCGTCGACCACCACCAGGACGTCGTTGTCGACGTCGGCCCCGCGGCTCCTGCCCGCCGAACGCGTCGTCGTCGACGGCCTGGTGCTCATCGACGAGCACCCGGAGCCAGGTGAAAAGCGCCGGCGGACGCTCGGACTGGGCGCGGGATTCCAGCAGTAACCCAGGTCCGCCGGCCACCTCACGGCAGGCAGACCCTCGGCTTTCGCGCCGGGACCGCCATGAGACCGCCACCCGGATGTCCGGGGTGGCGGTCTCATGGCGGTCCCTCAGCGGCCCTACGCGACCGCTGTGTCGACGCGGAGGCAGTCTCCGGGGTGCTGGCCCCAGGGCTGGAGTACCGGGGCGAGCTTGCCCTTGCAGCGGCGGCACACGGGCGACGCGGCGCCGGGCGGGGGAGTCTTGACGTCGACGGTGACCGGCTTCGGCTTCAGGCGCTTCCGAAGGCTGATCGTCGGGCGGATCGCCTCCTTCTTCGGCTGACGCACTCGGTCGAGCGAAGCGGCCAGCTCCTCCGTGCGGGCCTCGTTGGCCTTCCGGCGCGCCTCGCGGAAAGCAGCTTCCTCCTCGGACATGACCTCGAACCTCATCTGGTCAGCGTCAAGGTCGCCCGGCGCGGCCGGCGCTTCCGGGGCGGGCGGGTCCAGGACGTCCTTGCCCCACACCAGGCCCCAGGACTCGACGAGCCGCCGGACGCCCGGTAGGCCGTACGTCTCGGCGACCTTGATGAGGTCGAGGCGACGTCCGGCGACGCGGGCGATGTATCGGTACCAGATGTAGGCCGGGATGACCGCGATGGCGACCAGGCCCTCGGTGTCGTCGGTGATCTCCTCCTCGGTGCGGACGTCCTGCTGGATGCCGAGTTCCTTGATCAGCCGGTTCAGGTTCTGCGACCGGTAGTGCTTGCGGACCTGGAACACGCCGAACTCGCGCTCGCGGTACTTCTCGACGAACGGGCCGGGCCGACGAAGCCGCTGCAGCTCGGCGGCCGCCGCGTCGCCCAGGTCGAGCGGTCCCATGCGGTCGTCGCCGCGACCGGCCTTGAAGTTCTGTCCGGCCAGCTCCAGGCCGATCTTGGCGACGCCGCCCTTGGTCTTGTCGCCGTCCTTGTAGAGGTAGCGGGCCTGCTTGCCCGCATCGCCGTCAGCGGCGTCCGCGCCGTTGAGTGGGCGCACGTCGGTGCCGTGGCCCTTGCCCTCACAGGAGCAACCGGGCCGGTCGCACGTCTCGCTGACGGTGTAGCCGCCCGCGGATTCGACCCCGGCGGCCCAGGCTCCGGCGAGTGCGTCGCGGAACGCGGCCTGGGCGTCCGGGCCGAGCACCTCGCGGGTGACCCAGAGCGTGTGCCAGTGCAGGTGCCAGCCGGAGCCCCAGCCGAAGGTGTCCTCGAAGGCCCGCTCGTAGCCGATGATCCCGAAGTCGTCGCGCATCGTGCGCCAGCGGCGGCCGGACGAGCCGTACGCGCCCTTCCAGCCGTCGTGCAAGACCGCGACCAGGCCGTGCCGCATTCCCTTGCGGACGGTGCCGAACGCCATGCGCTCGAAGTGGCGCAACGTGTTCGTGCCAAGGTGCAGCCCGTACCCGGCGTCCGCGAGACCGTCGGCGGCGAGCTGCACGTTCGAGCCCCGTACGGCCAGGATGCGGCTCATGCACCACGGGCAGGTGTGGACGTTGTTGCAGCGGCACGTGTTGCCCCACGTCGCCTCGCCCGGCTTCCACATCAGCTCGGCCGTCCCGGCAGTGAGCCGGGTCCCGCAGCCCTTGAACGCCTCGTTCAGCGACACCGTCTGGTGCCGGTCCCGCCGGGCGAACCGCTCGTCGCGCGGGTCCTCCCGCCCGGCTGTCGCGGCACCCTCGTTTGGGGTAGAACCCGTTCCAGTTACAGCGCTCTGACCTGCAGTGGACGGAGATTTTCCCTTACTACTAAAGCCCGCGTCCGGATTACCCGCTGTAGTCGTGCTTGCTACGCTGCGTGACTGGTCCGCAATGAGACGCTTTGCGCGCTTTCGGCAGGCGTCCGAGCAGTAGATTTTGGGGCGCTTCCCGGGGATGTGGACGATCGGGGTGCCGCAGTGGCACTTCGGTCCGGCGGGGCGCGGTGGTGTCGACGCGCTGTTCTCTCGTACGCTCGTCACAGAGCAAACGTCCTCACTCGGCATGCTGCGCCGGTTCGGGGGCGGCGAGCCCGGGAGGCCAATCCCGGGCTCGTGCCATTTCTGGGTCCTGTTGATCCTGGCATTGGTGTGGCCGTTCATTGCCCCTGCTCGCTCCTGACGCGCCGATAGACGTCCGATACGCCCGGTGCTGGTGGGATTTGATAGGTCGGAAGAAGCCCCGCCCGGGCCTGGGCGGGGCTTCCTGTGCGTCAGGACCTCCTCGTCGTGAGCCTCTTCGGCCTATGGACGGAGTGACCTCGTGATCCGTTACAGCCGCGCGCGCTCGCGTAGAGCGGTCTCATCAGTTCCACGAACGGTCCTCTTCGCAGATCAGGGCGTTGGGGCGGAGTCTCACCAAGGACTACGTCTGCTGGCGATTTCCGTTACACCCCGGGCGGTGGCCGGCGCACACGCGCGCCCGCGTTGGGCAGTGCAGAAAGTGCAGAAACCTAGGCGCTGATGGTCCAGGTCCACGGTTCGTCGTCGGCGGCGGCGCGGGCGGCGGCGTCGGCCAGGGCGCGGGCGAGACCGGCTACGGCGGGCTTGATGCGCCGGTTGCGGGCGACCTTGAGCAGCTAGTATGCAGGTCGACGGATCTTTTCCGCTGCATAACCCTGCTTCGGGGTCATTATAGCGATTTTTTCGGTATATCCATCCTTTTTCGCACGATATACAGGATTTTGCCAAAGGGTTCGTGTAGACTTTCCTTGGTGTATCCAACGGCGTCAGCCGGGCAGGATAGGTGAAGTAGGCCCACCCGCGAGCGGGTGTTCCTTCTTCACTGTCCCTTATTCGCACCTGGCGGTGCTCAACGGGAATCCTGCTCTGCGAGGCTGGCCGGCTACCGCCGGCGTAACAGATGAGGGCAAGCGGATGGCTGATGAAACCAAGCCAACCAGGAAGGGCAGCCCACCTATCAAGGTGTACTGCCTTCCAGACGAACGAAGAGCGATTGAGGAAAAGGCGGCGGCGGCCGGCATGAGCCTGTCGGCCTACCTGCTGGCCGTCGGCCAGGGCTACAAAATCACGGGCGTCGTGGACTATGAGCACGTCCGCGAGCTGGACAGCGTGCAGGACTGGGGGAGTTA |
| **CRISPRa**  (Promoter-RiboJ-RBS-dCas9-XTEN linker - AD -terminator-promoter-sgRNA targeting sequence-sgRNA scaffold-terminator-ori-AprR-pSG5 rep-pSG5 ds-oriT) | TGTTCACATTCGAACCGTCTCTGCTTTGACATCGTGTGGCGCTTGGGTGTAAAGTCGTGGCCATTAAACAAAATTATTTGTAGAGGCTGTTTCGTCCTCACGGACTCATCAGACCGGAAAGCACATCCGGTGACAGCTAACTACGAAGGGGAGTCAGTATGGACAAGAAGTACAGCATCGGCCTGGCCATCGGCACCAACAGCGTGGGCTGGGCGGTCATCACCGACGAGTACAAGGTCCCCTCCAAGAAGTTCAAGGTCCTGGGCAACACCGACCGGCACTCGATCAAGAAGAACCTGATCGGCGCCCTGCTCTTCGACAGCGGCGAAACCGCCGAGGCGACCCGCCTGAAGCGGACCGCCCGTCGCCGCTACACCCGGCGCAAGAACCGCATCTGCTACCTGCAGGAGATCTTCTCCAACGAGATGGCCAAGGTCGACGACTCGTTCTTCCACCGGCTCGAGGAGAGCTTCCTGGTGGAGGAGGACAAGAAGCACGAGCGCCACCCGATCTTCGGCAACATCGTCGACGAGGTGGCCTACCACGAGAAGTACCCCACCATCTACCACCTCCGCAAGAAGCTGGTCGACTCGACCGACAAGGCGGACCTGCGGCTCATCTACCTGGCCCTCGCGCACATGATCAAGTTCCGCGGCCACTTCCTCATCGAGGGCGACCTGAACCCGGACAACTCCGACGTCGACAAGCTCTTCATCCAGCTGGTGCAGACCTACAACCAGCTGTTCGAGGAGAACCCCATCAACGCCAGCGGCGTCGACGCCAAGGCGATCCTCTCCGCGCGCCTGAGCAAGTCCCGGCGCCTGGAGAACCTCATCGCCCAGCTGCCGGGCGAGAAGAAGAACGGCCTCTTCGGCAACCTGATCGCGCTGTCGCTCGGCCTGACCCCCAACTTCAAGAGCAACTTCGACCTGGCCGAGGACGCGAAGCTCCAGCTGTCCAAGGACACCTACGACGACGACCTGGACAACCTGCTCGCCCAGATCGGCGACCAGTACGCGGACCTCTTCCTGGCCGCGAAGAACCTCTCGGACGCCATCCTGCTCAGCGACATCCTGCGGGTCAACACCGAGATCACCAAGGCCCCGCTGTCGGCGAGCATGATCAAGCGGTACGACGAGCACCACCAGGACCTGACCCTGCTCAAGGCCCTCGTGCGCCAGCAGCTGCCCGAGAAGTACAAGGAGATCTTCTTCGACCAGTCCAAGAACGGCTACGCCGGCTACATCGACGGCGGCGCGTCGCAGGAGGAGTTCTACAAGTTCATCAAGCCGATCCTGGAGAAGATGGACGGCACCGAGGAGCTGCTCGTCAAGCTGAACCGCGAGGACCTGCTCCGCAAGCAGCGGACCTTCGACAACGGCTCCATCCCGCACCAGATCCACCTGGGCGAGCTCCACGCCATCCTCCGGCGCCAGGAGGACTTCTACCCCTTCCTGAAGGACAACCGCGAGAAGATCGAGAAGATCCTGACCTTCCGGATCCCGTACTACGTCGGCCCCCTGGCCCGCGGCAACTCCCGGTTCGCGTGGATGACCCGGAAGTCGGAGGAAACCATCACCCCGTGGAACTTCGAGGAGGTCGTGGACAAGGGCGCCTCCGCGCAGTCGTTCATCGAGCGCATGACCAACTTCGACAAGAACCTCCCGAACGAGAAGGTCCTGCCCAAGCACAGCCTGCTCTACGAGTACTTCACCGTGTACAACGAGCTGACCAAGGTCAAGTACGTGACCGAGGGCATGCGGAAGCCGGCCTTCCTGTCCGGCGAGCAGAAGAAGGCGATCGTCGACCTGCTCTTCAAGACCAACCGCAAGGTCACCGTGAAGCAGCTGAAGGAGGACTACTTCAAGAAGATCGAGTGCTTCGACTCCGTCGAGATCTCGGGCGTGGAGGACCGCTTCAACGCCTCCCTGGGCACCTACCACGACCTGCTCAAGATCATCAAGGACAAGGACTTCCTCGACAACGAGGAGAACGAGGACATCCTGGAGGACATCGTCCTCACCCTGACCCTCTTCGAGGACCGCGAGATGATCGAGGAGCGGCTCAAGACCTACGCCCACCTGTTCGACGACAAGGTGATGAAGCAGCTGAAGCGGCGCCGGTACACCGGCTGGGGCCGCCTCTCCCGGAAGCTGATCAACGGCATCCGGGACAAGCAGAGCGGCAAGACCATCCTGGACTTCCTCAAGTCCGACGGCTTCGCCAACCGCAACTTCATGCAGCTCATCCACGACGACTCGCTGACCTTCAAGGAGGACATCCAGAAGGCCCAGGTGTCCGGCCAGGGCGACAGCCTCCACGAGCACATCGCCAACCTGGCGGGCTCCCCGGCGATCAAGAAGGGCATCCTCCAGACCGTCAAGGTCGTGGACGAGCTGGTCAAGGTGATGGGCCGCCACAAGCCCGAGAACATCGTGATCGAGATGGCCCGGGAGAACCAGACCACCCAGAAGGGCCAGAAGAACTCCCGCGAGCGGATGAAGCGCATCGAGGAGGGCATCAAGGAGCTCGGCTCGCAGATCCTGAAGGAGCACCCGGTCGAGAACACCCAGCTCCAGAACGAGAAGCTGTACCTCTACTACCTGCAGAACGGCCGCGACATGTACGTGGACCAGGAGCTCGACATCAACCGGCTGAGCGACTACGACGTCGACGCCATCGTGCCGCAGTCCTTCCTGAAGGACGACTCGATCGACAACAAGGTCCTGACCCGCTCCGACAAGAACCGGGGCAAGTCCGACAACGTGCCCTCGGAGGAGGTCGTGAAGAAGATGAAGAACTACTGGCGCCAGCTGCTCAACGCCAAGCTCATCACCCAGCGCAAGTTCGACAACCTGACCAAGGCCGAGCGGGGCGGCCTGTCGGAGCTCGACAAGGCGGGCTTCATCAAGCGCCAGCTCGTCGAAACCCGGCAGATCACCAAGCACGTGGCCCAGATCCTGGACAGCCGGATGAACACCAAGTACGACGAGAACGACAAGCTGATCCGCGAGGTCAAGGTGATCACCCTCAAGAGCAAGCTGGTGTCCGACTTCCGCAAGGACTTCCAGTTCTACAAGGTCCGGGAGATCAACAACTACCACCACGCCCACGACGCGTACCTGAACGCCGTCGTGGGCACCGCGCTGATCAAGAAGTACCCGAAGCTGGAGTCCGAGTTCGTCTACGGCGACTACAAGGTCTACGACGTGCGCAAGATGATCGCCAAGTCGGAGCAGGAGATCGGCAAGGCCACCGCGAAGTACTTCTTCTACAGCAACATCATGAACTTCTTCAAGACCGAGATCACCCTGGCCAACGGCGAGATCCGCAAGCGGCCCCTGATCGAAACCAACGGCGAAACCGGCGAGATCGTCTGGGACAAGGGCCGCGACTTCGCCACCGTCCGGAAGGTGCTGTCCATGCCGCAGGTCAACATCGTCAAGAAAACCGAGGTGCAGACCGGCGGCTTCAGCAAGGAGTCCATCCTCCCCAAGCGCAACTCGGACAAGCTGATCGCCCGGAAGAAGGACTGGGACCCGAAGAAGTACGGCGGCTTCGACAGCCCCACCGTCGCCTACTCCGTGCTGGTCGTGGCGAAGGTCGAGAAGGGCAAGAGCAAGAAGCTGAAGTCCGTGAAGGAGCTGCTCGGCATCACCATCATGGAGCGCTCCTCGTTCGAGAAGAACCCGATCGACTTCCTGGAGGCCAAGGGCTACAAGGAGGTCAAGAAGGACCTCATCATCAAGCTGCCCAAGTACTCGCTGTTCGAGCTCGAGAACGGCCGCAAGCGGATGCTCGCCAGCGCGGGCGAGCTGCAGAAGGGCAACGAGCTGGCCCTCCCGTCCAAGTACGTCAACTTCCTGTACCTCGCGTCCCACTACGAGAAGCTGAAGGGCTCGCCCGAGGACAACGAGCAGAAGCAGCTCTTCGTGGAGCAGCACAAGCACTACCTGGACGAGATCATCGAGCAGATCTCGGAGTTCAGCAAGCGGGTCATCCTGGCCGACGCGAACCTCGACAAGGTGCTGTCCGCCTACAACAAGCACCGCGACAAGCCGATCCGGGAGCAGGCGGAGAACATCATCCACCTGTTCACCCTCACCAACCTGGGCGCCCCCGCCGCGTTCAAGTACTTCGACACCACCATCGACCGCAAGCGGTACACCAGCACCAAGGAGGTCCTCGACGCGACCCTGATCCACCAGTCCATCACCGGCCTGTACGAAACCCGCATCGACCTCTCCCAGCTCGGCGGCGACAGTGGCTCAGAGACGCCGGGTACTTCTGAGTCCGCTACGCCTGAGagcATGCTTATCGCTCAGCGTCCTTCGCTGACCGAAGAGGTCGTCGACGAGTTCCGCTCCCGGTTCGTGATCGAGCCGCTGGAGCCGGGCTTCGGCTACACCCTCGGCAACTCCCTCCGCCGTACCCTCCTCTCCTCGATCCCGGGTGCCGCTGTCACCAGCATCCGCATCGACGGTGTCCTGCACGAGTTCACCACCGTGCCGGGCGTCAAGGAGGACGTCACCGACCTCATCCTCAACATCAAGCAGCTGGTCGTCTCCTCGGAGCACGACGAGCCGGTCGTGATGTACCTGCGCAAGCAGGGCCCGGGTCTGGTCACCGCCGCCGACATCGCGCCCCCGGCCGGTGTCGAGGTGCACAACCCCGACCTCGTCCTCGCCACGCTCAACGGCAAGGGCAAGCTGGAGATGGAGCTGACCGTCGAGCGCGGTCGCGGCTACGTCTCCGCCGTGCAGAACAAGCAGGTCGGTCAGGAGATCGGGCGCATCCCGGTCGACTCGATCTACTCGCCGGTTCTCAAGGTCACCTACAAGGTCGAGGCGACCCGAGTCGAGCAGCGCACCGACTTCGACAAGCTGATCGTCGACGTCGAGACCAAGCAGGCCATGCGCCCGCGTGACGCCATGGCGTCCGCCGGCAAGACCCTGGTCGAGCTGTTCGGTCTGGCGCGCGAGCTCAACATCGACGCCGAATTCAGATCTACGCGTTCCCCGCAAAAGCGGCCTTTGACTCCCTGCAAGCCTCAGCGACCGAATATATCGGTTATGCGTGGGCGATGGTTGTTGTCATTGTCGGCGCAACTATCGGTATCAAGCTGTTTAAGAAATTCACCTCGAAAGCAAGCTGATAAACCGATACAATTAAAGGCTCCTTTTGGAGCCTTTTTTTGACAGCGTGCAGGACTGGGGGAGTTATGTTCACATTCGAACCGTCTCTGCTTTGACACGGACAAGCGCTATGGTGTAAAGTCGTGGCCAcgtcagaattcacaagcccgGTTTTAGAGCTAGAAATAGCAAGTTAAAATAAGGCTAGTCCGTTATCAACTTGAAAAAGTGGCACCGAGTCGGTGCTTTTTACTCCATCTGGATTTGTTCAGAACGCTCGGTTGCCGCCGGGCGTTTTTTATCTAGAGGCCAGGAACCGTAAAAAGGCCGCGTTGCTGGCGTTTTTCCATAGGCTCCGCCCCCCTGACGAGCATCACAAAAATCGACGCTCAAGTCAGAGGTGGCGAAACCCGACAGGACTATAAAGATACCAGGCGTTTCCCCCTGGAAGCTCCCTCGTGCGCTCTCCTGTTCCGACCCTGCCGCTTACCGGATACCTGTCCGCCTTTCTCCCTTCGGGAAGCGTGGCGCTTTCTCATAGCTCACGCTGTAGGTATCTCAGTTCGGTGTAGGTCGTTCGCTCCAAGCTGGGCTGTGTGCACGACCCCCCCGTTCAGCCCGACCGCTGCGCCTTATCCGGTAACTATCGTCTTGAGTCCAACCCGGTAAGACACGACTTATCGCCACTGGCAGCAGCCACTGGTAACAGGATTAGCAGAGCGAGGTATGTAGGCGGTGCTACAGAGTTCTTGAAGTGGTGGCCTAACTACGGCTACACTAGAAGAACAGTATTTGGTATCTGCGCTCTGCTGAAGCCAGTTACCTTCGGAAAAAGAGTTGGTAGCTCTTGATCCGGCAAACAAACCACCGCTGGTAGCGGTGGTTTTTTTGTTTGCAAGCAGCAGATTACGCGCAGAAAAAAAGGATCTCAAGAAGATCCTTTGATCTTTTCTACGGGGTCTGACGCTCAGTGGAACGAAAACTCACGTTAAGGGATTTTGGTCATGAGATTATCAAAAAGGATCTTCACCTAGATCCTTTTGGTTCATGTGCAGCTCCATCAGCAAAAGGGGATGATAAGTTTATCACCACCGACTATTTGCAACAGTGCCGTTGATCGTGCTATGATCGACTGATGTCATCAGCGGTGGAGTGCAATGTCGTGCAATACGAATGGCGAAAAGCCGAGCTCATCGGTCAGCTTCTCAACCTTGGGGTTACCCCCGGCGGTGTGCTGCTGGTCCACAGCTCCTTCCGTAGCGTCCGGCCCCTCGAAGATGGGCCACTTGGACTGATCGAGGCCCTGCGTGCTGCGCTGGGTCCGGGAGGGACGCTCGTCATGCCCTCGTGGTCAGGTCTGGACGACGAGCCGTTCGATCCTGCCACGTCGCCCGTTACACCGGACCTTGGAGTTGTCTCTGACACATTCTGGCGCCTGCCAAATGTAAAGCGCAGCGCCCATCCATTTGCCTTTGCGGCAGCGGGGCCACAGGCAGAGCAGATCATCTCTGATCCATTGCCCCTGCCACCTCACTCGCCTGCAAGCCCGGTCGCCCGTGTCCATGAACTCGATGGGCAGGTACTTCTCCTCGGCGTGGGACACGATGCCAACACGACGCTGCATCTTGCCGAGTTGATGGCAAAGGTTCCCTATGGGGTGCCGAGACACTGCACCATTCTTCAGGATGGCAAGTTGGTACGCGTCGATTATCTCGAGAATGACCACTGCTGTGAGCGCTTTGCCTTGGCGGACAGGTGGCTCAAGGAGAAGAGCCTTCAGAAGGAAGGTCCAGTCGGTCATGCCTTTGCTCGGTTGATCCGCTCCCGCGACATTGTGGCGACAGCCCTGGGTCAACTGGGCCGAGATCCGTTGATCTTCCTGCATCCGCCAGAGGCGGGATGCGAAGAATGCGATGCCGCTCGCCAGTCGATTGGCTGAGCTCATGAGCGGAGAACGAGATGACGTTGGAGGGGCAAGGTCGCGCTGATTGCTGGGGCAACACGTGGAGCGGATCGGGGATTGTCTTTCTTCAGCTCGCTGATGATATGCTGACGCTCAATGCCGTTTGGCCTCCGACTAACGAAAATCCCGCATTTGGACGGCTGATCCGATTGGCACGGCGGACGGCGAATGGCGGAGCAGACGCTCGTCCGGGGGCAATGAGATATGAAAAAGCCTGAACTCACCGCGACGTATCGATGTCGAGGTTCCTCAGGGGAGCCACCCCAGAGAAGCCCTCGGAGCTGAGCGGAGCTATTTCCAAAGCCATGCCAGCTAGAGACAGTGCACACCGCCAAGCATCTGCAAAACCCTCGCCTGGAGGGAAAGTGCAATGTACGTACGCTGGTTTCCCTCCAGAAAGGGATTCTGCGGCTTCTCAACTTTGGAAGAAGAGGCGGAACGAACTGCTGTTTCAGCCTACTCATGTGAGGAGGCTGGTCCTTTACCCTGATGCCCGAGGGCATCAGGGTAAAGGACCAGCCTCGCCAACTAGGAGCGCTACCAAGGCGAACACAACCCAGGACTTGGTAGAACCCTTCGCCGGAGAAGTTCAGACTCATCGGCATGAGCACCCCTCGCCGCGCACTCGGCACCGGTCCGTCGACCACCACCAGGACGTCGTTGTCGACGTCGGCCCCGCGGCTCCTGCCCGCCGAACGCGTCGTCGTCGACGGCCTGGTGCTCATCGACGAGCACCCGGAGCCAGGTGAAAAGCGCCGGCGGACGCTCGGACTGGGCGCGGGATTCCAGCAGTAACCCAGGTCCGCCGGCCACCTCACGGCAGGCAGACCCTCGGCTTTCGCGCCGGGACCGCCATGAGACCGCCACCCGGATGTCCGGGGTGGCGGTCTCATGGCGGTCCCTCAGCGGCCCTACGCGACCGCTGTGTCGACGCGGAGGCAGTCTCCGGGGTGCTGGCCCCAGGGCTGGAGTACCGGGGCGAGCTTGCCCTTGCAGCGGCGGCACACGGGCGACGCGGCGCCGGGCGGGGGAGTCTTGACGTCGACGGTGACCGGCTTCGGCTTCAGGCGCTTCCGAAGGCTGATCGTCGGGCGGATCGCCTCCTTCTTCGGCTGACGCACTCGGTCGAGCGAAGCGGCCAGCTCCTCCGTGCGGGCCTCGTTGGCCTTCCGGCGCGCCTCGCGGAAAGCAGCTTCCTCCTCGGACATGACCTCGAACCTCATCTGGTCAGCGTCAAGGTCGCCCGGCGCGGCCGGCGCTTCCGGGGCGGGCGGGTCCAGGACGTCCTTGCCCCACACCAGGCCCCAGGACTCGACGAGCCGCCGGACGCCCGGTAGGCCGTACGTCTCGGCGACCTTGATGAGGTCGAGGCGACGTCCGGCGACGCGGGCGATGTATCGGTACCAGATGTAGGCCGGGATGACCGCGATGGCGACCAGGCCCTCGGTGTCGTCGGTGATCTCCTCCTCGGTGCGGACGTCCTGCTGGATGCCGAGTTCCTTGATCAGCCGGTTCAGGTTCTGCGACCGGTAGTGCTTGCGGACCTGGAACACGCCGAACTCGCGCTCGCGGTACTTCTCGACGAACGGGCCGGGCCGACGAAGCCGCTGCAGCTCGGCGGCCGCCGCGTCGCCCAGGTCGAGCGGTCCCATGCGGTCGTCGCCGCGACCGGCCTTGAAGTTCTGTCCGGCCAGCTCCAGGCCGATCTTGGCGACGCCGCCCTTGGTCTTGTCGCCGTCCTTGTAGAGGTAGCGGGCCTGCTTGCCCGCATCGCCGTCAGCGGCGTCCGCGCCGTTGAGTGGGCGCACGTCGGTGCCGTGGCCCTTGCCCTCACAGGAGCAACCGGGCCGGTCGCACGTCTCGCTGACGGTGTAGCCGCCCGCGGATTCGACCCCGGCGGCCCAGGCTCCGGCGAGTGCGTCGCGGAACGCGGCCTGGGCGTCCGGGCCGAGCACCTCGCGGGTGACCCAGAGCGTGTGCCAGTGCAGGTGCCAGCCGGAGCCCCAGCCGAAGGTGTCCTCGAAGGCCCGCTCGTAGCCGATGATCCCGAAGTCGTCGCGCATCGTGCGCCAGCGGCGGCCGGACGAGCCGTACGCGCCCTTCCAGCCGTCGTGCAAGACCGCGACCAGGCCGTGCCGCATTCCCTTGCGGACGGTGCCGAACGCCATGCGCTCGAAGTGGCGCAACGTGTTCGTGCCAAGGTGCAGCCCGTACCCGGCGTCCGCGAGACCGTCGGCGGCGAGCTGCACGTTCGAGCCCCGTACGGCCAGGATGCGGCTCATGCACCACGGGCAGGTGTGGACGTTGTTGCAGCGGCACGTGTTGCCCCACGTCGCCTCGCCCGGCTTCCACATCAGCTCGGCCGTCCCGGCAGTGAGCCGGGTCCCGCAGCCCTTGAACGCCTCGTTCAGCGACACCGTCTGGTGCCGGTCCCGCCGGGCGAACCGCTCGTCGCGCGGGTCCTCCCGCCCGGCTGTCGCGGCACCCTCGTTTGGGGTAGAACCCGTTCCAGTTACAGCGCTCTGACCTGCAGTGGACGGAGATTTTCCCTTACTACTAAAGCCCGCGTCCGGATTACCCGCTGTAGTCGTGCTTGCTACGCTGCGTGACTGGTCCGCAATGAGACGCTTTGCGCGCTTTCGGCAGGCGTCCGAGCAGTAGATTTTGGGGCGCTTCCCGGGGATGTGGACGATCGGGGTGCCGCAGTGGCACTTCGGTCCGGCGGGGCGCGGTGGTGTCGACGCGCTGTTCTCTCGTACGCTCGTCACAGAGCAAACGTCCTCACTCGGCATGCTGCGCCGGTTCGGGGGCGGCGAGCCCGGGAGGCCAATCCCGGGCTCGTGCCATTTCTGGGTCCTGTTGATCCTGGCATTGGTGTGGCCGTTCATTGCCCCTGCTCGCTCCTGACGCGCCGATAGACGTCCGATACGCCCGGTGCTGGTGGGATTTGATAGGTCGGAAGAAGCCCCGCCCGGGCCTGGGCGGGGCTTCCTGTGCGTCAGGACCTCCTCGTCGTGAGCCTCTTCGGCCTATGGACGGAGTGACCTCGTGATCCGTTACAGCCGCGCGCGCTCGCGTAGAGCGGTCTCATCAGTTCCACGAACGGTCCTCTTCGCAGATCAGGGCGTTGGGGCGGAGTCTCACCAAGGACTACGTCTGCTGGCGATTTCCGTTACACCCCGGGCGGTGGCCGGCGCACACGCGCGCCCGCGTTGGGCAGTGCAGAAAGTGCAGAAACCTAGGCGCTGATGGTCCAGGTCCACGGTTCGTCGTCGGCGGCGGCGCGGGCGGCGGCGTCGGCCAGGGCGCGGGCGAGACCGGCTACGGCGGGCTTGATGCGCCGGTTGCGGGCGACCTTGAGCAGCTAGTATGCAGGTCGACGGATCTTTTCCGCTGCATAACCCTGCTTCGGGGTCATTATAGCGATTTTTTCGGTATATCCATCCTTTTTCGCACGATATACAGGATTTTGCCAAAGGGTTCGTGTAGACTTTCCTTGGTGTATCCAACGGCGTCAGCCGGGCAGGATAGGTGAAGTAGGCCCACCCGCGAGCGGGTGTTCCTTCTTCACTGTCCCTTATTCGCACCTGGCGGTGCTCAACGGGAATCCTGCTCTGCGAGGCTGGCCGGCTACCGCCGGCGTAACAGATGAGGGCAAGCGGATGGCTGATGAAACCAAGCCAACCAGGAAGGGCAGCCCACCTATCAAGGTGTACTGCCTTCCAGACGAACGAAGAGCGATTGAGGAAAAGGCGGCGGCGGCCGGCATGAGCCTGTCGGCCTACCTGCTGGCCGTCGGCCAGGGCTACAAAATCACGGGCGTCGTGGACTATGAGCACGTCCGCGAGCTGGACAGCGTGCAGGACTGGGGGAGTTA |

Supplementary table 3. Promoters used in this study. Plasmid sequences can be constructed by replacing the yellow regions in the example plasmids shown above. Promoter sequences were derived from Bai et al.^1^, Myronovskyi and Luzhetskyy^2^, and Phelan et al^3^.

| **Promoter ID** | **Sequence (5’ to 3’)** |
| --- | --- |
| 57 | TTGAACGGCTGGAGGGATACACCTGGTCATAGGATACCATC |
| ermE*p | CTCTAGTATGCATGCGAGTGTCCGTTCGAGTGGCGGCTTGCGCCCGATGCTAGTCGCGGTTGATCGGCGATCGCAGGTGCACGCGGTCGATCTTGACGGCTGGCGAGAGGTGCGGGGAGGATCTGACCGACGCGGTCCACACGTGGCACCGCGATGCTGTTGTGGGCACAATCGTGCCGGTTGGTAGGATCCACAT |
| gapdh(EL) | GCTGCTCCTTCGGTCGGACGTGCGTCTACGGGCACCTTACCGCAGCCGTCGGCTGTGCGACACGGACGGATCGGGCGAACTGGCCGATGCTGGGAGAAGCGCGCTGCTGTACGGCGCGCACCGGGTGCGGAGCCCCTCGGCGAGCGGTGTGAAACTTCTGTGAATGGCCTGTTCGGTTGCTTTTTTTATACGGCTGCCAGATAAGGCTTGCAGCATCTGGGCGGCTACCGCTATGATCGGGGCGTTCCTGCAATTCTTAGTGCGAGTATCTGAAAGGGGATACGC |
| KasO*p | TGTTCACATTCGAACGGTCTCTGCTTTGACAACATGCTGTGCGGTGTTGTAAAGTCGTGGCC |
| rpsl(XC) | GCCCTGCAGGCGGAAGTCAGGTAGACACGACTTCCGCTAGTCCTTGCAAGGTCTGCTGACGTGAGGCGGGGCGGTCGTTTTTGACCGCCCTGCCTTCGTCATGTAGGCTCGCTCGCTGTGCCTGGCGTGTCATCAGACGCCCAGGTCCCGGTGCCGTGAGGCCCGGGCCATCGAGCCGGTGGTACGTGGCTGCGGTCCCCTTGTGAGGGCTGCGCGCCGTGTGCTGTCCGGCGCGCACAGCCTTGAATCCACCCGCGGGGGCCGGCCGGTCTCCGTGAGCTCGAGTAGACGACGGAGACGTA |
| SP1 | TGTTCACATTCGAACCGTCTCTGCTTTGACATGGAGAGAAGTTTTGTAAAGTCGTGGCCA |
| SP10 | TGTTCACATTCGAACCGTCTCTGCTTTGACATGTTCTTACGGTCACATGTAAAGTCGTGGCCA |
| SP20 | TGTTCACATTCGAACCGTCTCTGCTTTGACACATGACGCTCACCCGTTGTAAAGTCGTGGCCA |
| SP30 | TGTTCACATTCGAACCGTCTCTGCTTTGACATCGTGTGGCGCTTGGGTGTAAAGTCGTGGCCA |
| SP43 | TGTTCACATTCGAACCGTCTCTGCTTTGACACGGACAAGCGCTATGGTGTAAAGTCGTGGCCA |

Supplementary table 4. sgRNA sequences used in this study. Plasmid sequences can be constructed by replacing the dark yellow regions in the example plasmids shown above.

| **Sequence**  **(5’ to 3’)** | **PAM location** | **Strand** | **Target** | **Type** | **Tool** | **Plasmid** |
| --- | --- | --- | --- | --- | --- | --- |
| CATGTTGTCCTCCTCGCCCT | 11 | NT | mCherry | On-target | CRISPRi | pJEC711, pJEC712, pJEC713, pJEC714 |
| TCTGGGTGCCCTCGTACGGC | 123 | NT | mCherry | On-target | CRISPRi | pJEC727 |
| GTCGGCCGGGTGCTTGACGT | 230 | NT | mCherry | On-target | CRISPRi | pJEC728 |
| TCTTGACTTCGGCGTCGTAG | 531 | NT | mCherry | On-target | CRISPRi | pJEC729 |
| CGTGTAGTCCTCGTTGTGCG | 623 | NT | mCherry | On-target | CRISPRi | pJEC730 |
| CAAGGGCGAGGAGGACAACA | 29 | T | mCherry | On-target | CRISPRi | pJEC731 |
| GGGGAGGGCCGGCCGTACGA | 132 | T | mCherry | On-target | CRISPRi | pJEC732 |
| GAAGGCCTACGTCAAGCACC | 242 | T | mCherry | On-target | CRISPRi | pJEC733 |
| CGAAGTCAAGACGACGTACA | 560 | T | mCherry | On-target | CRISPRi | pJEC734 |
| CAACGAGGACTACACGATCG | 647 | T | mCherry | On-target | CRISPRi | pJEC735 |
| GGCTCAGGTGAAGAGCGGGG | NA | NT | Non-coding genomic sequence | Off-target | CRISPRi, CRISPRa | pJEC723, pJEC737, pJEC740, pJEC743 |
| GCGTGCGTCGCACCTCCGTG | NA | NT | Non-coding genomic sequence | Off-target | CRISPRi | pJEC724 |
| TGCACTGCTGTAAGGACGAT | NA | NA | No match | Off-target | CRISPRi | pJEC725 |
| CCGTGTGGCCCGCTCTTGTT | 44 | NT | jadR2 | On-target | CRISPRi | pJEC749 |
| AGGTATCCTGCGGTGTCCTG | -82 | T | mCherry | On-target | CRISPRa | pJEC739, pJEC742, pJEC745 |
| AGGACGCCTTTGGTAACCGC | -83 | NT | mCherry | On-target | CRISPRa | pJEC738, pJEC741, pJEC744 |
| ATGTGAACAAAGGACGCCTT | -73 | NT | mCherry | On-target | CRISPRa | pJEC747 |
| TGGTAACCGCAGGACACCGC | -93 | NT | mCherry | On-target | CRISPRa | pJEC748 |
| CGTCAGAATTCACAAGCCCG | -73 | NT | jadJ | On-target | CRISPRa | pJEC750 |

Supplementary table 5. Activator domains (ADs) used in this study. Plasmid sequences can be constructed by replacing the dark green regions in the example plasmids shown above.

| **AD** | **Nucleotide sequence** | **Protein sequence** | **Plasmids** |
| --- | --- | --- | --- |
| αNTD | ATGCTTATCGCTCAGCGTCCTTCGCTGACCGAAGAGGTCGTCGACGAGTTCCGCTCCCGGTTCGTGATCGAGCCGCTGGAGCCGGGCTTCGGCTACACCCTCGGCAACTCCCTCCGCCGTACCCTCCTCTCCTCGATCCCGGGTGCCGCTGTCACCAGCATCCGCATCGACGGTGTCCTGCACGAGTTCACCACCGTGCCGGGCGTCAAGGAGGACGTCACCGACCTCATCCTCAACATCAAGCAGCTGGTCGTCTCCTCGGAGCACGACGAGCCGGTCGTGATGTACCTGCGCAAGCAGGGCCCGGGTCTGGTCACCGCCGCCGACATCGCGCCCCCGGCCGGTGTCGAGGTGCACAACCCCGACCTCGTCCTCGCCACGCTCAACGGCAAGGGCAAGCTGGAGATGGAGCTGACCGTCGAGCGCGGTCGCGGCTACGTCTCCGCCGTGCAGAACAAGCAGGTCGGTCAGGAGATCGGGCGCATCCCGGTCGACTCGATCTACTCGCCGGTTCTCAAGGTCACCTACAAGGTCGAGGCGACCCGAGTCGAGCAGCGCACCGACTTCGACAAGCTGATCGTCGACGTCGAGACCAAGCAGGCCATGCGCCCGCGTGACGCCATGGCGTCCGCCGGCAAGACCCTGGTCGAGCTGTTCGGTCTGGCGCGCGAGCTCAACATCGACGCC | MLIAQRPSLTEEVVDEFRSRFVIEPLEPGFGYTLGNSLRRTLLSSIPGAAVTSIRIDGVLHEFTTVPGVKEDVTDLILNIKQLVVSSEHDEPVVMYLRKQGPGLVTAADIAPPAGVEVHNPDLVLATLNGKGKLEMELTVERGRGYVSAVQNKQVGQEIGRIPVDSIYSPVLKVTYKVEATRVEQRTDFDKLIVDVETKQAMRPRDAMASAGKTLVELFGLARELNIDA | pJEC737, pJEC738, pJEC739, pJEC747, pJEC748, pJEC750 |
| ω | GTGTCCTCTTCCATCACCGCGCCCGAGGGCATCATCAACCCGCCGATCGACGAGCTGCTCGAGGCCACGGACTCGAAGTACAGCCTCGTGATCTACGCGGCCAAGCGCGCGCGTCAGATCAACGCGTACTACTCCCAGCTCGGCGAGGGCCTGCTGGAGTACGTGGGTCCGCTGGTCGACACCCACGTCCACGAGAAGCCGCTCTCGATCGCCCTGCGCGAGATCAACGCGGGCCTGCTGACGTCCGAGGCCATCGAGGGCCCGGCGCAG | VSSSITAPEGIINPPIDELLEATDSKYSLVIYAAKRARQINAYYSQLGEGLLEYVGPLVDTHVHEKPLSIALREINAGLLTSEAIEGPAQ | pJEC740, pJEC741, pJEC742 |
| RbpA | ATGAGTGAGCGAGCTCTTCGCGGCACGCGCCTCGTGGTGACGAGCTACGAGACCGACCGCGGCATCGATCTGGCCCCGCGCCAGGCCGTGGAGTACGCATGCGAGAAGGGCCATCGTTTTGAGATGCCCTTCTCGGTGGAAGCGGAAATTCCGCCGGAGTGGGAGTGCAAGGTCTGCGGAATCCAGGCACTCCTGGTGGACGGGGACGGACCTGAGGAGAAGAAGGGCAAGCCTGCGCGTACGCACTGGGACATGCTCATGGAGCGACGCACCCGCGAGGAGCTGGAGGAGGTCCTCGCCGAAAGGCTGGCCGTCCTGCGTTCCGGCGCCATGAACATCGCCGTGCATCCGCGCGACAGCCGCAAGTCCGCC | MSERALRGTRLVVTSYETDRGIDLAPRQAVEYACEKGHRFEMPFSVEAEIPPEWECKVCGIQALLVDGDGPEEKKGKPARTHWDMLMERRTREELEEVLAERLAVLRSGAMNIAVHPRDSRKSA | pJEC743, pJEC744, pJEC745 |

**Supplementary figure 1. CRISPRi can repress transcription in *Streptomyces*.** A plasmid expressing dCas9 and a sgRNA that targets the coding sequence of mCherry was conjugated into a *S. venezuelae* strain expressing a genomically integrated mCherry reporter. Fluorescence characterization was performed by bulk fluorescence measurements (measured in units of fluorescence [FL]/optical density [OD] at 600 nm). Data represent mean values and errors bars represent standard deviation of 4 biological replicates. Statistical significance was calculated using a two-tailed unpaired Welch’s t-test. Asterisks represent a p-value lower than 0.001.

Supplementary figure 2. Evaluating the strength of a library of *Streptomyces* promoters. Fluorescence characterization of constitutive promoters. The expression strength of a constitutive promoter library was evaluated by cloning each promoter upstream of an mCherry reporter and integrating the resulting reporter constructs into the genome of *S. venezuelae* cells at the ΦC31 attB site. Fluorescence characterization was performed by bulk fluorescence measurements (measured in units of fluorescence [FL]/optical density [OD] at 600 nm). Data represent mean values and errors bars represent standard deviation of 4 biological replicates.

Supplementary figure 3. CRISPRi results in decreased fluorescence in the absence of a sgRNA or in the presence of non-targeting sgRNA. Fluorescence characterization of *S. venezuelae* cells containing a genomically-integrated mCherry gene conjugated with CRISPRi plasmids containing different sgRNAs or a no CRISPRi control plasmid. mCherry sgRNA binds to the mCherry gene and represses expression. Genomic sequence 1 and 2 are sgRNAs designed to non-coding sequences present in the *S. venezuelae* genome. Non-targeting sgRNA is designed to target a sequence absent in the *S. venezuelae* genome. No sgRNA is a CRISPRi plasmid without a sgRNA. Fluorescence characterization was performed by bulk fluorescence measurements (measured in units of fluorescence [FL]/optical density [OD] at 600 nm). Data represent mean values and errors bars represent standard deviation of 3 biological replicates.

**Supplementary figure 4. Evaluating distance-dependent activation patterns of CRISPRa.** Fluorescence characterization of *S. venezuelae* cells containing a genomically-integrated mCherry gene conjugated with CRISPRa plasmids containing different sgRNAs or a no CRISPRa control plasmid. Blank cells are *S. venezuelae* cells lacking mCherry and transformed with the no CRISPRa plasmid that are used to determine autofluorescence. In (a) a reporter containing PAMs on the template strand at 73, 83, and 93 bp upstream of the reporter promoter’s TSS is used. In (b) an additional 5 bp are added before the reporter promoter’s TSS to create PAMs at 78 and 88 bp upstream. Fluorescence characterization was performed by bulk fluorescence measurements (measured in units of fluorescence [FL]/optical density [OD] at 600 nm). Data represent mean values and errors bars represent standard deviation of 4 biological replicates

**Supplementary figure 5. Ion count-based estimation of jadomycin B produced using CRISPRi.** **a**. LC-MS analysis of crude extracts of *S. venezuelae* cells conjugated with CRISPRi plasmids designed to repress the expression of jadR2. Cells were cultured, fermented, and extracted as described in methods. The crude extracts were then analyzed via liquid chromatography coupled to mass spectrometry (LC-MS). The reported data are multiple reaction monitoring (MRM) chromatograms at m/z 550.2 → 420.1 ([M+H]+). The Y axis shows the ion abundance. A jdB standard was run in parallel, and showed the same elution time as the CRISPRi sample. **b**. Area under the curve.

Supplementary figure 6. Additional replicates of the jadomycin B production experiment using CRISPRi. a. LC-MS analysis of crude extracts of *S.* *venezuelae* cells conjugated with CRISPRi plasmids designed to repress the expression of jadR2. Cells were cultured, fermented, and extracted as described in methods. The crude extracts were then analyzed via liquid chromatography coupled to mass spectrometry (LC-MS). The reported data are extracted ion chromatogram at the corresponding *m/z* value of jdB (m/z = 550.2059, [M+H]^+^) for a second set of representative biological replicates (other than the ones shown in Figure 3c). The Y axis shows the absorbance intensity. A jdB standard was run in parallel, and showed the same elution time as the CRISPRi sample. We note that the observed elution time of these replicates differs from the data shown in Figure 3c due to different instrumentation. For these samples, LC-MS analysis was carried out using a Shimadzu IT-ToF MS system interfaced to a Shimadzu LC-20 ADXR LC system through an Electro Sprat Ionization (ESI) source. Separations were carried out using an Ascentis Express C18 column (1.0 mm ID x 150 mm with 90A, 2.7u particles). The LC was operated at a flow rate of 0.175 mL/min with mobile phase A (0.1% Formic Acid in Water) and B (0.1% Formic Acid in Acetonitrile). The Analytical separation was carried out over 10 min; going from 10%B (t= 0 min) to 95% B(t= 10 min). b. Area under the curve.

**Supplementary figure 7. Ion count-based estimation of jadomycin B produced using CRISPRa.** **a.** LC-MS analysis of crude extracts of strains harboring jadJ-V-targeting CRISPRa plasmids. To induce the production of jdB, a sgRNA was designed to target a sequence downstream of a PAM site at 73 bp from the TSS of the jadJ-V operon within the jdB BGC. The sgRNA was cloned into a plasmid harboring the αNTD-based CRISPRa system. The plasmid was conjugated into wild-type *S. venezuelae*, and the resulting strains were cultured, fermented, and extracted. The crude extracts were then analyzed via liquid chromatography coupled to mass spectrometry (LC-MS), as described in the methods. The reported data are multiple reaction monitoring (MRM) chromatograms at m/z 550.2 → 420.1 ([M+H]+) for a second set of representative biological replicates (other than the ones shown in Figure 3d). A jdB standard was run in parallel, and showed the same elution time as the CRISPRa sample. **b.** Area under the curve.

Supplementary figure 8. Additional replicates of the jadomycin B production experiment using CRISPRa. a. LC-MS analysis of crude extracts of strains harboring jadJ-V-targeting CRISPRa plasmids. To induce the production of jdB, a sgRNA was designed to target a sequence downstream of a PAM site at 73 bp from the TSS of the jadJ-V operon within the jdB BGC. The sgRNA was cloned into a plasmid harboring the αNTD-based CRISPRa system. The plasmid was conjugated into wild-type *S. venezuelae*, and the resulting strains were cultured, fermented, and extracted. The crude extracts were then analyzed via liquid chromatography coupled to mass spectrometry (LC-MS), as described in the methods. The reported data are multiple reaction monitoring (MRM) chromatograms at m/z 550.2 → 420.1 ([M+H]+) for a second set of representative biological replicates (other than the ones shown in Figure 3d). The Y axis shows the ion abundance. A jdB standard was run in parallel, and showed the same elution time as the CRISPRa sample. b. Area under the curve.

**REFERENCES**

1. Bai, C. *et al.* Exploiting a precise design of universal synthetic modular regulatory elements to unlock the microbial natural products in Streptomyces. *Proc. Natl. Acad. Sci.* **112**, 12181–12186 (2015).

2. Myronovskyi, M. & Luzhetskyy, A. Native and engineered promoters in natural product discovery. *Nat. Prod. Rep.* **33**, 1006–1019 (2016).

3. Phelan, R. M. *et al.* Development of next generation synthetic biology tools for use in streptomyces venezuelae. *ACS Synth. Biol.* **6**, 159–166 (2017).
